# High-Quality Draft Genome Sequence of *Xanthomonas* sp. Strain CPBF 424, a Walnut-Pathogenic Strain with Atypical Features

**DOI:** 10.1128/MRA.00921-18

**Published:** 2018-10-18

**Authors:** Camila Fernandes, Jochen Blom, Joël F. Pothier, Fernando Tavares

**Affiliations:** a CIBIO—Centro de Investigação em Biodiversidade e Recursos Genéticos, InBIO Laboratório Associado, Microbial Diversity and Evolution Group, Universidade do Porto, Vairão, Portugal; b Instituto Nacional de Investigação Agrária e Veterinária, Unidade Estratégica de Investigação e Serviços de Sistemas Agrários e Florestais e Sanidade Vegetal, Oeiras, Portugal; c Faculdade de Ciências, Departamento de Biologia, Universidade do Porto, Porto, Portugal; d Bioinformatics and Systems Biology, Justus-Liebig-University Giessen, Giessen, Germany; e Environmental Genomics and Systems Biology Research Group, Institute for Natural Resource Sciences, Zurich University of Applied Sciences (ZHAW), Wädenswil, Switzerland; Louisiana State University

## Abstract

We report here the draft genome sequence of *Xanthomonas* sp. strain CPBF 424, isolated from a diseased walnut tree. Multilocus sequence analysis showed that this walnut-pathogenic isolate is located between the nonpathogenic X. arboricola and X. prunicola clusters. These features make this strain a promising reference to disclose new genetic determinants of pathogenesis.

## Abstract (precis)

We report here the draft genome sequence of *Xanthomonas* sp. strain CPBF 424, isolated from a diseased walnut tree.

## ANNOUNCEMENT

The Xanthomonas arboricola species complex includes numerous phytopathogenic bacteria comprising different pathovars capable of infecting a wide range of plants (1–3) and causing severe disease symptoms and serious economic losses in important crops (4). Recently, particular attention has been given to X. arboricola-related strains shown to be phylogenetically distinct from pathogenic X. arboricola pathovar strains (5–8). *Xanthomonas* sp. strain CPBF 424 was isolated in April 2016 from asymptomatic dormant buds of a diseased walnut tree in Loures, Portugal, with common symptoms of walnut bacterial blight. Multilocus sequence analysis (MLSA) of the concatenated partial sequences of the *atpD* (750 bp), *dnaK* (759 bp), *efp* (339 bp), *fyuA* (684 bp), *glnA* (675 bp), *gyrB* (735 bp), and *rpoD* (586 bp) genes confirmed the strain’s identity as a *Xanthomonas* sp., revealing that strain CPBF 424 is located between the nonpathogenic X. arboricola and X. prunicola clusters and diverges from Xanthomonas arboricola pv. juglandis strains, i.e., walnut-pathogenic bacteria, and from other X. arboricola pathovars (9). Pathogenicity tests on walnut plantlets further showed that CPBF 424 is pathogenic to walnut trees (10, 11), making this strain particularly appealing to provide new insights into xanthomonad pathoadaptations.

Here, we make available the whole-genome sequence of *Xanthomonas* sp. strain CPBF 424.

*Xanthomonas* sp. strain CPBF 424 was grown on bacterial culture medium M2 (yeast extract, 2 g liter^−1^; Bacto peptone, 5 g liter^−1^; NaCl, 5 g liter^−1^; KH_2_PO_4_, 0.45 g liter^−1^; Na_2_HPO_4_ 12H_2_O, 2.39 g liter^−1^) at 28°C and 100 rpm for 48 h. DNA was extracted for sequencing using the E.Z.N.A. bacterial DNA purification kit (Omega Bio-tek, Norcross, GA). Genomic library preparation and genome sequencing were outsourced to GATC Biotech, AG (Konstanz, Germany) and conducted using an Illumina HiSeq platform with 2 × 150-bp paired-end reads, which resulted in 12,672,550 reads of raw sequence data with a sequencing coverage of 776×. *De novo* genome assembly was obtained with MIRA version 4.0 (12) using standard settings in accurate mode. This was followed by contig reassembly using SeqMan Pro from the Lasergene genomics package version 12.1.0 (DNAStar, Madison, WI) with Pro assembler parameters and read mapping using SeqMan NGen with standard settings to check for inconsistencies (overlapping contig extremities with no or low coverage with paired-read inconsistencies). A total of five irregularities were found on which contigs were broken open per the initial *de novo* assembly. Contigs were ordered using the Move Contigs function in Mauve 20150226 version 10 (13, 14) according to the genome of X. arboricola pv. juglandis CFBP 2528 (GenBank accession number NZ_JZEF00000000) (15). Automatic genome annotation was performed with a *Xanthomonas* genus database using the Prokka software tool version 1.12 (16).

The *Xanthomonas* sp. CPBF 424 genome had a total size of 4,896,146 bp and a G+C content of 65.89% represented by 10 contigs with an *N*_50_ value of 1,029,447 bp. The genome of CPBF 424 is estimated to be composed of 4,143 coding sequences (CDS), including 58 tRNAs and 4 rRNAs. Preliminary analysis with the EDGAR version 2.0 platform (17) allowed us to detect 3,502 coding sequences that are shared between CPBF 424 and X. arboricola pv. juglandis CFBP 2528, which was used as the reference genome.

The whole-genome sequence of strain CPBF 424 may contribute to elucidating new walnut pathoadaptations within the genus *Xanthomonas*.

### Data availability.

This whole-genome shotgun project has been deposited in DDBJ/ENA/GenBank under the BioProject accession number PRJEB27248 (SRA accession number ERR2767968), and the sequence accession number is UIHB00000000. The version described in this paper is version UIHB01000000.
